# Voice vs. silence: the role of cognitive appraisal of and emotional response to stressors

**DOI:** 10.3389/fpsyg.2023.1079244

**Published:** 2023-04-25

**Authors:** Phoebe Haemin Pahng, Sung Mo Kang

**Affiliations:** ^1^Business Management, James Madison University, Harrisonburg, VA, United States; ^2^Economics and Business Department, Cornell College, Mount Vernon, IA, United States

**Keywords:** stressor, employee voice, appraisal, emotion, prospect theory, threat-rigidity

## Abstract

Stress is in the nature of work, employees, teams, and organizations. Some speak up under stress, whereas others keep silent. Given that employee voice has long been recognized to enhance high-quality decisions and organizational effectiveness, understanding conditions under which employees practice voice is important. In this article, we combine appraisal theory, prospect theory, and threat-rigidity thesis so as to enrich our understanding of the relationship between stressors and voice. In so doing, our theory paper integrates threat-rigidity thesis, prospect theory, and appraisal theory on the basis of the interaction between cognition and emotion, and it explores the detailed cognition-emotion-behavior (voice) relationship.

## 1. Introduction

W*hy* and *when* would some employees speak up in response to workplace stressors, whereas others stay silent? Here, stressors refer to agents/conditions that require sustained cognitive, emotional, and/or physical attention (De Jonge and Dormann, 2006); and speaking up (i.e., voice) refers to “any attempt at all to change, rather than escape from an objectionable state of affairs” (Hirschman, 1970, p.30). Given that employee voice has long been recognized to enhance high-quality decisions and organizational effectiveness (Burris, 2012; Morrison, 2014; Chamberlin et al., 2017), understanding the conditions under which employees practice voice has been considered important in the literature. To this point, a wide range of key conditions of employee voice has been empirically identified, such as psychological safety (e.g., Liang et al., 2012), a supportive climate to encourage speaking up (e.g., Frazier and Fainshmidt, 2012; Wang and Hsieh, 2013), and high quality of exchange relationship with leaders (e.g., Detert and Burris, 2007; Detert and Treviño, 2010).

Despite the rapid progress in voice research through understanding the antecedents of employee voice (for reviews and meta-analytic evidence, see Morrison, 2011, 2014; Chamberlin et al., 2017), the relationship between stressors and voice remains unclear (e.g., Xia et al., 2020). This can be due to the oversimplification of the relationship by assuming that individuals in stressful situations respond similarly. For example, Ng and Feldman (2012) argue that employees are less likely to speak up under job and social stressors in order to protect and retain personal resources (e.g., time, energy, and attention).

However, cognitive and emotional responses to a stressor, not the stressor itself, constitute a reality that may affect an individual’s subsequent behavior (Lewin, 1936). On the one hand, prospect theorists (e.g., Kahneman and Tversky, 1979) would argue that workplace stressors are positively associated with employee voice, as individuals perceiving the potential losses are more risk-seeking (i.e., employee voice) than those perceiving the potential gains. On the other hand, proponents of threat-rigidity perspective (e.g., Staw et al., 1981) would contend that workplace stressors are negatively associated with employee voice, since individuals perceiving threats exhibit rigidity, or rigid/avoidance behaviors by freezing information processing and constraining challenging decision-making or behaviors. To explain such seemingly contradictory predictions of the association between stressors and voice, we maintain that considering the impact of individuals’ cognitive and emotional responses to stressors on voice is essential, as reactions to stressors may vary by individual. In so doing, we explore prospect theory, threat rigidity theory, and appraisal theory to address the stressor-voice relationship.

Accordingly, studying cognitive and emotional responses to stressors is important in understanding stressor-voice relationship. Yet, studies regarding the impact of cognition and emotion on the stressor-voice relationship are relatively rare. Only two studies (Zhang et al., 2014; Long et al., 2015) investigated the impact of appraisal of stressors (i.e., cognition) on voice. Also, relatively few studies have investigated the association between emotion and voice (e.g., Ashford et al., 1998; Grant, 2013). The results, yet, do not directly address the issue of *why* and *when* stressors positively or negatively affect voice. The purpose of this study, then, is to explore the relationship between stressor and voice. Here, we argue that individual differences in cognitive and emotional response to stressors account for differences in whether employees decide to speak up or not.

**FIGURE 1:**
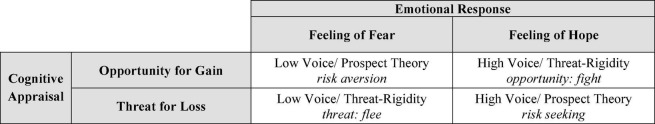
Cognitive and emotional response to stressors.

For this purpose, the article contributes to existing theories as well as organizational behavior literature. First, it contributes to employee voice literature. This paper can be a response to Crant’s (2000) call for examining the role of cognitive processes in decisions to engage in proactive behavior (i.e., employee voice). Yet, this paper moves a step forward and considers emotional responses and cognitive appraisals to predict voice. Thus, this study contributes to employee voice literature by clarifying *why* the effect of stressors on employee voice varies and understanding how to encourage employee voice to increase organizational effectiveness. Second, this article contributes to the existing appraisal theory. One of the main criticism regarding appraisal theory is that it lacks explicit theories pertaining to the expected appraisal-emotion-behavior association (e.g., Zhan, 2020). Drawing on threat-rigidity thesis and prospect theory, this paper addresses the concern and predicts how cognition and emotion can separately or interactively shape behaviors of individuals. Finally, this article has a theoretical contribution as it integrates threat-rigidity perspective and prospect theory. That is, a potential contradiction between prospect theory and threat-rigidity perspective discussed above may be resolved by considering different combinations of appraisals and emotions (see Figure 1).

In the remainder of the article, this study reviews extant literature regarding stress-voice relationship, provides a theoretical framework for this study, develops our propositions on the basis of prospect theory and threat-rigidity perspective, and discusses future directions regarding our suggested model.

## 2. Stressor-voice association

### 2.1. Stressors

Stressors are defined as conditions or environmental events of threats, challenges, and demands that require sustained cognitive, emotional, and/or physical attention (De Jonge and Dormann, 2006). Stressors are different from stress, which is oftentimes conceptualized as an interactive process between an individual and threats, challenges, and constraints of their environment (i.e., stressors) that causes a change in mental or physical health (Folkman and Lazarus, 1985; Ganster and Rosen, 2013). That is, stress is an outcome of stressors. As the focus of this article is to examine different responses to a condition that requires cognitive, emotional, and/or physical attention, this study assumes stressor as a pre-condition to cognitive and emotional response.

According to Ng and Feldman (2012), three types of stressors exist-job stressors, social stressors, and organizational stressors-depending on the sources of those stressors. Job stressors refer to attributes of the work itself that require sustained cognitive, emotional, or physical effort on the part of employees (De Jonge and Dormann, 2006). Examples of job stressors include time pressures, high levels of responsibility, and perceptions of lack of job challenge (Cavanaugh et al., 2000; Ng and Feldman, 2012). Social stressors refer to interpersonal relationships that require sustained cognitive, emotional, or physical effort on the part of employees (De Jonge and Dormann, 2006). Examples of social stressors include strained relationships with supervisors, supervisor interactional unfairness, and strained relationships with coworkers (Ng and Feldman, 2012). Organizational stressors refer to stimuli in the broader organizational environment that demand sustained cognitive, emotional, or physical effort on the part of employees (De Jonge and Dormann, 2006). Examples of organizational stressors include organizational politics, breaches of promises or expectations, distributive/procedural unfairness, lack of organizational support, and lack of organizational communication (Ng and Feldman, 2012).

The consequences of stressors in workplace have been studied by many scholars, such as employee performance and well-being outcomes (Rodell and Judge, 2009; Rosen et al., 2020). Such relationships have been dominantly explained through the challenge and hindrance stressor framework introduced by Cavanaugh et al. (2000). Challenge stressor refers to the perception of a stressor as having potential for growth, mastery, and/or gain; whereas hindrance stressor refers to the perception of a stressor as having potential for harm or loss (Folkman and Lazarus, 1985). In general, challenge stressors are positively and significantly associated with performance, whereas hindrance stressors are negatively and significantly associated with performance (Lepine et al., 2005).

### 2.2. Voice

Employees differ in their subsequent behaviors following stressors-some may engage in proactive behavior, while others may not. Proactive employees act in advance rather than react, and take actions to change themselves and/or the situation (Grant and Ashford, 2008; Lebel, 2017). Speaking up to those who are able to change the situation, selling issues to top management, taking charge or initiative by introducing new policies, procedures, or practices, and seeking feedback about performance and job status are common forms of proactive behavior studied in the field of organizational behavior (Crant, 2000; Morrison, 2014). This study particularly focuses on voice, which refers to “informal and discretionary communication by an employee of ideas, suggestions, concerns, information about problems, or opinions about work-related issues to persons who might be able to take appropriate action, with the intent to bring about improvement or change” (Van Dyne and LePine, 1998; Morrison, 2014, p. 174). The employee voice is also conceptualized as a constituent of job performance as it represents the most challenging form of citizenship behavior (Zhang et al., 2014). In addition, according to Zhang et al. (2014), stressor-job performance relationship requires more nuanced examination as the association may differ by the operationalization of job performance. Hence, we focus on voice behavior as one of job performance.

Employee voice entails general actions pertaining to proactive behavior such as identifying opportunities to improve things, challenging the *status quo*, and creating favorable conditions (Morrison, 2011). However, despite such a constructive motive, employee voice inherently accompanies social risks since it can seem like challenges to a leader or organization (Burris, 2012; Fast et al., 2014), and voicer can be viewed or labeled negatively (e.g., a complainer or troublemaker) (Milliken et al., 2003; Morrison, 2011). Accordingly, voice entails a risk of going beyond the job requirements as well as going beyond what management wants their employees to do (Frese and Fay, 2001; Parker et al., 2006). Although employee voice is inherent risk-taking behavior, it can also bring many benefits to individuals, teams, and organizations. For example, certain types of voice have been significantly associated with positive performance evaluation, liking, attribution of prosocial behavior, and so on (Whiting et al., 2008; Morrison, 2014). In addition, empirical evidence suggests that groups and organizations perform better and have increased learning and less turnover when employees express ideas, opinions, and concerns (Spencer, 1986; De Dreu and West, 2001; Van Dyne et al., 2003; Morrison, 2014; Chamberlin et al., 2017).

### 2.3. Stressors and voice

The stressor-voice association is important to investigate because there are reasons to expect both positive and negative relationships between these two constructs (Ng and Feldman, 2012). On the one hand, prospect theorists (e.g., Kahneman and Tversky, 1979; Tversky and Kahneman, 1992; Rieger and Wang, 2006) would argue that workplace stressors are positively associated with employee voice, because employees are loss averse. More specifically, prospect theory argues that individuals tend to be averse to risks when facing gains but to seek risks when facing losses. Indeed, Seo et al. (2010) found support that loss (appraisal) is related to risk-taking behavior.

On the other hand, proponents of threat-rigidity perspective (e.g., Staw et al., 1981) would contend that workplace stressors are negatively associated with employee voice, because employees’ tendency to act rigidly (i.e., threat rigidity) under stressful conditions. In other words, based on threat-rigidity perspective, individuals in the face of adversity or threats stop considering challenging behaviors or something new to change or improve current situations since they feel more comfortable maintaining the *status quo* and try to converse diminishing resources. Such an argument is supported by Chattopadhyay et al. (2001) as they found that organizational actions tend to be internally directed under threat (loss) conditions. Because two countervailing arguments can be made in regard to stressors-voice association, it is important to investigate the theoretical nature of the association, which potentially resolves the paradox.

However, research examining how stressors are related to voice has been limited, and what little has been done has reported mixed results (e.g., Long et al., 2015). Pertaining to stressors-voice association, Ng and Feldman (2012) conducted a meta-analytic study, and found support for the negative association between work stressors and voice. Although the authors, indeed, extended our knowledge regarding the stressor-voice relationship, several limitations to this study require further examination of the relationship. That is, the authors used conservation of resource theory (Hobfoll, 1989) in predicting the direction of the association, and they argued that this theory could predict both positive and negative association between stressor and voice. This indicates that conservation of resource theory may be limited in predicting stressor-voice relations. Further, they conceptualize a stressor as a negative condition (e.g., lack of job autonomy, strained relationships with supervisors, distributive unfairness), when, in reality, a stressor can be appraised as a positive condition (e.g., an opportunity for gain) as well (e.g., Cavanaugh et al., 2000). Thus, this study provides an alternative theoretical framework (i.e., prospect theory and threat rigidity thesis) to explain both positive and negative associations between stressor and voice.

## 3. Theoretical framework

In situations that are highly ambiguous, appraisal of stressors as well as emotional responses to stressors can differ by employees (Folkman and Lazarus, 1985). This is due to the difficulty in evaluating the likely outcomes pertaining to stressors (Folkman and Lazarus, 1985). First, some employees may appraise stressors as an opportunity, whereas others may appraise them as a threat (e.g., Cavanaugh et al., 2000). Second, some employees may feel fear under stressors; whereas others may feel hope under stressors (Folkman and Lazarus, 1985; Lebel, 2017). Lazarus (1993) maintains that such appraisals and emotions can affect behaviors differently. For example, threat appraisals and negative emotions can be related to avoidance behavior (Lazarus, 1993). Based on previous literature (e.g., Campbell and Jones, 2002; Gomes et al., 2017), we argue that cognitive and emotional responses to stressor, not the stressor itself, affect voice.

Given that stressors may be perceived differently by employees, and that employees may feel different emotions regarding those stressors, we use prospect theory and threat-rigidity perspective so as to link stressors and voice with the following reasons. First, both threat-rigidity perspective and prospect theory are closely related to cognitive appraisal of and emotional response to stressors, as both approaches consider appraisal and emotion in predicting behavior. That is, both theories predict proactive/risk-taking behavior on the basis of cognition and emotion, although both views seem to emphasize cognition. In discussing threat-rigidity, Staw et al. (1981) provided examples of cognitive appraisals (e.g., perception of stress) as well as emotional responses (e.g., anxiety) that affect rigid behaviors. In discussing prospect theory, Tversky and Kahneman (1981) elaborated on how emotional responses (i.e., displeasure) interact with cognitive appraisals (i.e., gain or loss) to predict individuals’ risk-taking behavior. Second, both theories do not assume rationality in decision-making. That is, both views modify the expected utility model and no longer assume that people are rational decision-makers. Third, both theories apply to individual unit-level constructs, although threat-rigidity perspective considers group-level and organizational-level constructs as well.

Nevertheless, at the surface level, threat-rigidity perspective and prospect theory seem to contradict each other (Chattopadhyay et al., 2001). On the one hand, threat-rigidity perspective (Staw et al., 1981) mainly suggests that individuals, when they appraise situation as a threat, tend to behave rigidly (i.e., tendency toward well-learned or dominant response). According to Staw et al. (1981), the underlying mechanisms of this argument are the restriction of information processing and constriction in control. That is, a stressor may result in restriction of information processing, like a reduction in the number of channels used in decision-making. Further, stressors may result in a constriction of control, like power being concentrated in higher levels of a hierarchy. Together, these mechanisms affect employees to act rigidly when they experience stressors. On the other hand, prospect theory (Tversky and Kahneman, 1992) mainly suggests that employees, when they appraise a stressor as a threat, tend to take more risks. According to Holmes et al. (2011), the main mechanism behind this argument is individual’s tendency to be loss averse. Specifically, when employees appraise a stressor as a threat, they feel that they have nothing to lose, which then leads them to become risk-seekers. Hence, on the basis of threat-rigidity perspective and prospect theory, both positive and negative associations between stressors and voice can be predicted.

**FIGURE 2:**
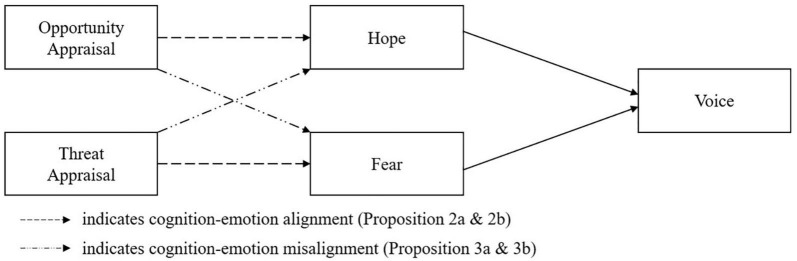
The proposed conceptual model.

Such a potential paradox, however, can be resolved when cognition and emotion as well as the interaction between the two, are taken into consideration. Here, we argue that threat-rigidity perspective can better predict stressor-voice association when cognition and emotion are aligned, whereas prospect theory can better predict stressor-voice association when cognition and emotion are not aligned. According to appraisal theory (Folkman and Lazarus, 1985), threat appraisals are linked to threat emotions (i.e., fear, anxiety, and worry), while opportunity appraisals are linked to opportunity emotions (i.e., hope, eagerness, and confidence). Although the threat-rigidity perspective does not explicitly discuss cognition-emotion alignment, it seems to work similarly to appraisal theory as a mechanism that mediates stressors and voice, and the predicted outcomes are similar to that of appraisal theory. That is, both appraisal theory and threat-rigidity regard constriction of control to be the underlying mechanism that links stressors and voice. Further, both perspectives predict that avoidance can result from threat appraisals (Staw et al., 1981; Lazarus, 1993). On the contrary, prospect theory assumes a misalignment between cognition and emotion. For example, prospect theory predicts that opportunity appraisal is associated with fear because individuals feel that they have more to lose when they perceive stressors as an opportunity. The comparisons of the theories are presented in Table 1. In the following sections, we discuss in detail how two different views can predict voice, considering cognition and emotion. Figure 2 describes our conceptual model.

**TABLE 1:** Summary/comparison of appraisal theory, threat rigidity, and prospect theory.

	Appraisal theory (Folkman and Lazarus, 1985)	Threat-rigidity thesis (Staw et al., 1981)	Prospect theory (Kahneman and Tversky, 1976)
Level of analysis	Individual	Individual, group, organizational	Individual
Cognition	Threat/opportunity appraisal	Threat	Loss/gain framing
Emotion	Threat emotions (e.g., fear, anxiety, worry)/ Opportunity emotions (e.g., hope, eagerness, confidence)	Anxiety	Displeasure/pleasure
Mechanism	Coping	Constriction of control, restriction in information processing	Loss aversion
Outcome	Threat-avoidance	Threat: flee/ Opportunity: fight	Loss: risk-seeking Gain: risk-aversion

## 4. Theory development

### 4.1. Emotion and voice

An individuals’ reactions to job events often bypass cognitive appraisal and directly impact emotional responses (Weiss et al., 1999). Folkman and Lazarus (1985) categorized fear/anxiety/worry as threat emotions and hope/confidence/eagerness as opportunity emotions. We argue that both prospect theory and threat-rigidity thesis may apply in predicting emotion-voice relationship. That is, both threat-rigidity thesis and prospect theory would predict a negative association between fear and voice, whereas both theories would predict a positive association between hope and voice. Further, mechanisms relevant to both theories-constriction of control and loss aversion-may apply. One important study regarding the association between affect and proactive behavior is Lebel’s (2017) conceptual work, which provides a model that links negative emotions (i.e., fear and anger) to proactive behavior. Regarding fear, the author argues that fear could be positively associated with proactive behavior when individuals perceive fear as manageable.

Taking the discrete emotions perspective (i.e., a perspective on emotions as discrete, not aggregate), this study focuses on fear and hope as emotional responses to stressors. This is because both sets of feelings of fear and hope are known to arise out of uncertainty but differ in their focus on positive (opportunity/gain) or negative (threat/loss) outcomes (Roseman, 2011; Lebel, 2017). Individuals hesitate to speak up and keep silent when they feel fear since fear is a powerful emotion to increase withdrawal response and habituated schema-based behavior (e.g., not speaking up to managers) (Kish-Gephart et al., 2009). However, individuals who feel positive emotions are more likely to have more controllability, and broader perspective of the context, and a sense of safety in the working environment (Watson, 2000). Indeed, empirical evidence supports this proposition as fear was found to be positively related to withdrawal (Ashford et al., 1989; Rafferty and Griffin, 2006) and silence (Milliken et al., 2003). In addition, Madrid (2020) has found that positive emotions and feelings encourage employees to speak up. Thus, this study argues that fear is negatively associated with voice, but hope is positively associated with voice. Employee’s displeasure associated with loss attenuates when they feel hope, but employees may increase withdrawal response and maintain their habitual behaviors when they feel fear.

*Proposition 1. Employees are less likely to speak up following emotional response to a stressor as fearful, but they are more likely to speak up following emotional response to a stressor as hopeful.*

### 4.2. Cognition-emotion alignment

As stated above, stressors can be appraised as a threat or an opportunity. According to Folkman and Lazarus (1985) the cognitive appraisal of stressors includes two component processes, primary and secondary appraisal. Through primary appraisal, individuals decide whether the condition is relevant or irrelevant to one’s well-being. Once individuals evaluate the condition (i.e., stressor) as being “relevant,” they then evaluate whether the stressor would be an opportunity or a threat. Here, an opportunity refers to the potential for growth, mastery, or gain; whereas a threat refers to the potential for harm and loss. Notably, subjective, not objective, appraisal of stressors is what matters. Appraisal theorists (Folkman and Lazarus, 1985; Lazarus, 1993) posit that opportunity appraisal and threat appraisal are associated with opportunity emotions (i.e., hope, eagerness, and confidence) and threat emotions (i.e., fear, anxiety, and worry), respectively. As such, alignment between cognition (i.e., appraisal) and emotion is an important assumption of appraisal theory.

The underlying mechanism and outcome predicted by appraisal theory are very similar to those of threat-rigidity perspective. First, both appraisal theory and threat-rigidity perspective consider controllability as an underlying mechanism that links appraisal/emotion and behavior. Second, the outcome associated with threat appraisal and control is avoidance or rigidity that are similar to each other. That is, appraisal theory (Folkman and Lazarus, 1985; Lazarus, 1993) suggests that, after the primary appraisal discussed above, individuals move on to the secondary appraisal (but two types of appraisals, primary and secondary, frequently occur simultaneously; Lazarus and Folkman, 1984), where they evaluate coping resources and options, addressing the question, “What can I do?” Such secondary appraisal is similar to the control mechanism discussed by Staw et al. (1981). According to the authors, rigid behavior entails constriction of control where individuals feel that they have little control over the situation. Second, both threat-rigidity perspective and appraisal theory predict that when individuals appraise the stressors as a threat without possessing the necessary resource to deal with them, individuals’ constriction of control occurs, which leads to rigid/avoidance behavior (Staw et al., 1981). Thus, when cognition aligns with emotions, threat-rigidity may explain the nature of the stressor-voice relationship.

As stated above, the threat rigidity perspective posits that there is a general tendency for individuals, teams, and organizations to behave rigidly (i.e., a tendency toward well-learned or dominant response) in threatening conditions. Such predictions may be valid as long as emotions align with the appraisals as discussed above. Given that employee voice involves individuals initiating change and taking a risk by behaving beyond one’s role, threat rigidity theory predicts that individuals are less likely to practice voice when they appraise the situation as a loss and they experience fear due to stressors. Conversely, individuals who appraise stressors as an opportunity and experience hope following stressors, he/she is more likely to speak up. This is because individuals are more likely to experience constriction of control (i.e., a feeling that he/she has less control over the situation) under the threat conditions, whereas they are likely to feel more control over the situation in the opportunity context.

On the basis of the above arguments, we propose that.

*Proposition 2a. Employees are less likely to speak up in response to the interaction between the cognitive appraisal of a stressor as a threat and the emotional response to a stressor as fearful. The more the employee perceives a stressor as a threat for loss, and the more the employee feels fear, the less the employee will speak up.*

*Proposition 2b. Employees are more likely to speak up in response to the interaction between the cognitive appraisal of a stressor as an opportunity for gain and the emotional response to a stressor as hopeful. The more the employee perceives a stressor as an opportunity for gain, and the more the employee feels hope, the more the employee will speak up.*

### 4.3. Cognition-emotion misalignment

What if appraisals and emotions do not align? Some scholars have argued that cognition precedes emotion (e.g., Folkman and Lazarus, 1985), whereas others have argued that emotion precedes cognition (e.g., Swinyard, 1993; Rolls, 2000). Further, some scholars have argued that cognition and emotion are interdependent with each other (e.g., Folkman and Lazarus, 1985; Storbeck and Clore, 2007), whereas others have argued that cognition and emotion are independent of each other (Zajonc, 2000). Instead of taking one perspective over the other, this study takes a comprehensive approach regarding the relationship between cognition and emotion. In the previous section, this study used the appraisal theory and threat-rigidity thesis to explain situations where emotions and appraisals align; this is based on the interdependence approach. However, in this section, we consider the possibility that cognition and emotion are independent of each other. Accordingly, this study assumes that cognitive appraisal and emotional response can occur separately and can interact with each other in making an impact on behavior. In this case, we argue that prospect theory can be used to predict voice behavior under situations where cognition and emotion do not align.

Indeed, prospect theory (Tversky and Kahneman, 1992) helps clarify the interaction effect of cognition and emotion on risk-taking behavior when there is a misalignment between appraisal and emotion. In prospect theory, outcomes are expressed as positive or negative deviations (gains or losses) from a neutral reference outcome. Notably, one property of prospect theory is that the response to losses is more extreme than the response to gains. According to Tversky and Kahneman (1981) such differences in responses to gain and loss are explained with displeasure associated with losing money that is greater than the pleasure associated with winning the money. Although Tversky and Kahneman put a lot of emphasis on cognitive appraisal of the situation in predicting behavior, both cognition (appraisal of opportunity/gain and threat/loss) and emotion (pleasure/displeasure) are the driving forces that distance outcomes from the neutral reference outcome. The importance of emotion in prospect theory is posited by Mercer (2005) as well. Simply put, prospect theory (implicitly) posits that when individuals appraise the condition as a loss, they feel that they have nothing to lose, and in turn their fear related to loss goes away. As such, prospect theory assumes that fear related to stressors may decrease or hope related to stressors may increase under threat conditions. Hence, we use prospect theory in predicting voice behavior when cognition and emotions do not match.

The underlying mechanism of prospect theory is an individual’s tendency to loss aversion, meaning that they find displeasure associated with loss to be greater than pleasure associated with gain (Holmes et al., 2011). For this reason, the value function is concave above the reference point and convex below it. Such a pattern contributes to risk-seeking preferences for individuals involving only losses and risk-averse preferences for those involving only gains. However, such an argument only applies to situations where cognition and emotions do not align (i.e., threat appraisal-opportunity emotions and opportunity appraisal-threat emotions).

Notably, we are also categorizing appraisal and no-emotion association as a misalignment between cognition and emotion, because individuals would generally experience emotions in accordance with cognition. Employees who appraise the situation as a threat are likely to feel fear. Yet, a no-emotion condition delineates a situation where employees do not feel fear even when they appraise the situation as a threat. Hence, misalignment conditions constitute threat-hope, opportunity-fear, threat-no-emotion, and opportunity-no-emotion conditions. In those cases, we use prospect theory to predict stressor-voice relationship. When cognitions and emotions are misaligned, prospect theory can predict voice. This is due to loss aversion.

On the basis of the above arguments, this study proposes that.

*Proposition 3a. Employees are less likely to speak up in response to the interaction between the cognitive appraisal of a stressor as an opportunity for gain and the emotional response to a stressor as fearful. The more the employee perceives a stressor as an opportunity for gain, and the more the employee feels fear, the less the employee will speak up.*

*Proposition 3b. Employees are more likely to speak up in response to the interaction between the cognitive appraisal of a stressor as a threat of loss and the emotional response to a stressor as hopeful. The more the employee perceives a stressor as a threat of loss, and the more the employee feels hope, the more the employee will speak up.*

## 5. Discussion

### 5.1. Theoretical contributions

First, this study contributes to employee voice literature by examining cognitive and emotional processes that potentially link stressors and voice. Especially, Crant (2000) called for the development of theories that tie together antecedents and proactive behavior using cognitive processes. By examining emotional responses along with cognitive appraisals to predict voice, this paper moves a step forward from Crant (2000)’s suggestion. Also, this paper attempts to resolve a previously unresolved issue regarding proactive behavior – the role of environmental change. Specifically, this paper argues that it is not the change itself, but an employee’s cognitive and emotional responses to change, that predict an employee’s proactive behavior (i.e., employee voice).

Second, this study contributes to appraisal theory by complementing the theory with threat rigidity thesis and prospect theory. One of the main criticisms regarding appraisal theory is that it lacks explicit theories about the expected cognition-emotion-behavior relationship (Zhan, 2020). Drawing from threat-rigidity thesis and prospect theory, this paper addresses the concern and predicts how cognition as well as emotion can separately or interactively impact proactive behavior. Although traditional appraisal theory assumes that cognitive appraisal of an event is associated with emotional response to an event, appraisal theorists disagree on *how* and *why* cognitions and emotions are related (Fernando et al., 2017). In this paper, we take the comprehensive approach to cognition and emotion, and posit that certain appraisals and emotions arise in response to stressors. That is, due to the uncertainty associated with stressors, threat-opportunity appraisals as well as threat-opportunity emotions occur. Prospect theory implies that such cognitions and emotions may occur independently, while threat-rigidity thesis implies that such cognitions and emotions are interdependent. In this study, we try to complement traditional appraisal theory, and argue that particular associations and the effects of those patterns can predict voice behavior differently, using threat-rigidity and prospect theory.

Third, this study has a theoretical contribution by integrating threat-rigidity perspective and prospect theory, which are contradictory at the surface level. Threat-rigidity perspective posits that individuals become rigid (risk-averse) in threat conditions, whereas prospect theory posits that individuals become risk-seekers in threat conditions. This study attempts to resolve such contradiction by considering different dynamics played by cognitions and emotions in response to stressors. Both threat-rigidity thesis and prospect theory consider framing (i.e., appraisal) of an emotional response to a situation in order to predict behaviors. However, the threat-rigidity perspective is more relevant to examining the effect of cognition and emotion when they align with each other, whereas prospect theory is more concerned with the interaction between cognition and emotion when they do not align. Hence, by separating cognition and emotion and examining different combinations pertaining to those two, we integrate threat-rigidity perspective and prospect theory.

### 5.2. Limitations and directions for future research

It is important to recognize several limitations pertaining to the scope of this study. First, although many of our arguments are based on previous empirical research, this study does not empirically test our theoretical model. Most importantly, we encourage additional empirical research on our propositions and the overall theory and framework we have proposed. To help future research, we suggest two types of empirical research designs to test our proposed model. First, future research could use scenario-based experiments (a total of nine conditions) to manipulate experimental conditions consisting of 3 appraisals (opportunity, threat, no appraisal) and 3 emotions (hope, fear, no emotion). The scenario-based experimental designs would allow future research to test different paths of opportunity/threat appraisals, hope/hear emotion, and voice behavior in terms of the three theories (i.e., threat-rigidity thesis, prospect theory, appraisal theory) we suggested. It, then, helps us understand the causal relationships between cognitions, emotions, and voice behavior. Second, scholars could utilize an experience sampling methodology (ESM) to collect daily survey data from employees to measure daily appraisals, emotions, and voice behaviors and test their relationships. The primary advantage of ESM studies is the ability to simultaneously test relationships between- and within-level for the same participants (Gabriel et al., 2019). Since employees appraise and respond to work-related events differently on a daily basis, many organizational studies have already utilized ESM approach to study employee emotions and voice behaviors (e.g., Dimotakis et al., 2011; Liu et al., 2022). This approach would allow future research to investigate the dynamic relationships among cognitions, emotions, and voice behavior.

Second, this study focuses on, and thus, is limited to, cognitive and emotional responses to employee voice. Accordingly, individual differences (e.g., personality, risk-taking propensity, self-efficacy, gender), social factors (e.g., social support), and cultural factors (e.g., cultural genes) that have been studied to affect employee voice are not considered in this study. For example, individuals with high risk-taking propensity are more likely to speak up when they perceive stressors as a threat for loss and then feel fear, but individuals with low risk-taking propensity might show a different pattern of behaviors. In this case, risk-taking propensity would moderate the relationship between stressors and voice. In addition, cultural differences delineate and impact how individuals perceive work environments and stressors, which determine their linguistic expressions such as speaking up and being silent (Pishghadam et al., 2020). Thus, future studies may investigate how individual, situational, and cultural factors interact with cognitive and emotional processes discussed in the current study.

## 6. Conclusion

“If we accept arguments that have been made in practitioner outlets that proactive behavior is more crucial than ever because of the changing nature of work as we enter the 21st century, it is important for researchers to further specify the process by which people decide whether or not to engage in proactive behaviors and ways to engage in proactive behaviors more effectively” (Crant, 2000, p.459). In line with this argument, we examine *when* and *why* some speak up in response to stressors, whereas others do not. In so doing, we attempt to integrate potentially contradictory perspectives: prospect theory and threat-rigidity perspective. On the one hand, when cognition and emotion are aligned (e.g., threat-fear, opportunity-hope), we contend that we can use threat rigidity framework to predict voice behaviors. On the other hand, when cognition and demotion are misaligned (e.g., threat-hope, opportunity-fear), we argue that we should use prospect theory and predict voice behaviors. In sum, we propose that employees tend to speak up under opportunity-hope and threat-hope conditions, whereas they tend to be silent under oopportunity-fear and threat-fear conditions. This study contributes to voice literature by exploring the dynamics played between cognition and emotion, so as to link stressors and voice.

## Author contributions

PP designed this study. Both authors built the study framework, wrote the manuscript, contributed to the article, and approved the submitted version.
